# Pediatric Laparoscopic Surgery in Pakistan

**Published:** 2016-01-01

**Authors:** Bilal Mirza

**Affiliations:** Department of Pediatric Surgery, The Children’s Hospital and the Institute of Child Health, Lahore

In Pakistan laparoscopic surgery is not new but its application in pediatric surgery has been very limited. In the last decade, impalpable undescended testes mainly; and occasionally appendicitis and cholecystitis were few maladies dealt with laparoscopy in only two or three pediatric surgery units of the country. Very few publications were brought out from Pakistan. The pediatric surgery residents have not been trained or given adequate exposure to the modality during their training.

In the last 5 years, few pediatric surgery units took initiative to provide hands on training to the pediatric surgeons and residents. At Children’s Hospital Lahore, workshop was conducted for residents and junior consultants for learning techniques of gaining access and port insertion. The most important of all the activities were two workshops; one conducted in April 2015 at Karachi (Liaquat National Hospital) arranged through BAPS Hugh Greenwood grant (International B.A.P.S Hugh Greenwood Laparoscopy Skills Course); and other conducted recently in December 2015 at National Institute of Child Health Karachi, a live video conference on advanced pediatric laparoscopic surgery. These workshops provided very meaningful exposure and skills of basic and advanced laparoscopy to the attending pediatric surgeons from various parts of the country. The general laparoscopic surgeons have also contributed to inspire and train their pediatric surgery counterparts.

The current lot of pediatric surgeons is very enthusiastic for learning and practicing pediatric laparoscopic surgery. Many junior and senior consultants are acquiring laparoscopic skills and majority of pediatric surgery units located in Lahore, Karachi, Nawabshah, Islamabad, Multan, Bahawalpur etc. are now practicing pediatric laparoscopic surgery though only few are doing advanced laparoscopic procedures in children e.g. repair of congenital diaphragmatic hernia and eventration, laparoscopic assisted anorectoplasty, pyloromyotomy, fundoplication, Wilm’s tumor excision, VATS for empyema thoracic and hydatid cyst, cystic abdominal lesions, germs cell tumors etc. are few in the list. Few Pediatric surgeons have completed fellowship in Pediatric Laparoscopic Surgery from China. Some pediatric surgeons returned back to the country after acquiring skills from western countries and doing marvelous work. Formation of an association of pediatric laparoscopic surgeons of Pakistan is being deemed. The future of pediatric laparoscopic surgery looks promising in Pakistan. With maturation of learning curve, more advanced procedures and research will be seen from this part of world.

## Footnotes

**Source of Support:** Nil

**Conflict of Interest:** None declared

